# Reporting incidence from a surveillance system with an operational case definition of unknown predictive value positive

**DOI:** 10.1186/1742-5573-2-7

**Published:** 2005-07-20

**Authors:** Scott R Kegler

**Affiliations:** 1 Office of Statistics and Programming, National Center for Injury Prevention and Control, Centers for Disease Control and Prevention, 4770 Buford Highway NE, Mailstop K59, Atlanta GA 30341-3724, USA

## Abstract

When reporting incidence rate estimates for relatively rare health conditions, associated case counts are often assumed to follow a Poisson distribution. Case counts obtained from large-scale electronic surveillance systems are often inflated by the presence of false positives, however, and adjusted case counts based on the results of a validation sample will have variances which are hyper-Poisson. This paper presents a simple method for constructing interval estimates for incidence rates based on case counts that are adjusted downward using an estimate of the predictive value positive of the surveillance case definition.

## Introduction

Large-scale surveillance for selected medical or health conditions often relies on electronic data sources which provide comprehensive coverage of a given population. For example, the Centers for Disease Control and Prevention conduct surveillance of brain injuries involving hospitalization or death, based on electronic hospital discharge and vital statistics data received from twelve to fifteen states each year [1]. To identify cases, electronic records are scanned for specified diagnosis codes which collectively form the operational case definition. The resulting case counts are subsequently combined with population data to estimate incidence rates.

As with most surveillance methods, an operational case definition as described above may admit some records that do not represent true cases under a strict clinical definition ("false positives") and may also fail to capture some records representing true cases ("false negatives"). The customary terms reflecting these aspects of an operational case definition are predictive value positive (PVP) and sensitivity, defined in the present context as the conditional probabilities [2]:

PVP = Pr{case meets clinical definition | case detected under operational definition};

sensitivity = Pr{case detected under operational definition | case meets clinical definition}.

Depending on the extent to which false positives and/or false negatives are believed to influence the surveillance process, it may be appropriate to use estimates of PVP and/or sensitivity to adjust incidence rate estimates accordingly. It is not generally possible to assess PVP or sensitivity using electronic surveillance data alone. The most direct approach to obtaining the additional data required for estimation of PVP involves manual review of medical records for a random sample of provisional cases identified via the operational case definition. Obtaining the additional data necessary for estimation of sensitivity may be more labor-intensive, particularly when considering an uncommon condition. Without additional "markers" (apart from the operational case definition) to narrow the scope of review, it may be necessary to select a very large sample of general medical records in order to identify enough true cases to support a stable estimate of sensitivity.

The methodology described in this paper is oriented to surveillance of relatively rare health conditions. Because validation data quantifying the influence of false positives will typically be easier to obtain than data quantifying the influence of false negatives in this setting, the development concentrates on incidence rate estimates reflecting adjustments for PVP. This emphasis is not intended to diminish the potential influence of false negatives; rather, it reflects the logistical difficulties associated with obtaining data on false negatives as part of ongoing surveillance. If there is sufficient doubt surrounding the sensitivity of case ascertainment for any particular surveillance process, the proposed methodology should be applied with due caution.

## Analysis

For a given surveillance period, it is assumed that case confirmation data are available for a random sample (selected without replacement) of provisional cases. Data obtained through such validation efforts allow estimation of PVP as well as adjustments to case counts to eliminate the bias due to false positives. To illustrate, suppose that for a set period (e.g., one year) of observation:

N = size of the at-risk population covered by the surveillance system;

M = count of provisional cases detected under the operational case definition;

M_T _= count of true cases (unknown) among the provisional cases;

M_F _= count of false positive cases (unknown) among the provisional cases = M - M_T_;

S = number of provisional cases sampled for case confirmation;

C_T _= count of confirmed true cases among those sampled;

C_F _= count of cases determined to be false positives among those sampled = S - C_T_.

The usual estimate of PVP is given by [3]:

 = C_T_/S = C_T_/(C_T _+ C_F_).

Noting that  is definable only when M > 0 (assuming also that S > 0) a reasonable estimate of the population of true cases which eliminates the false positive bias is:



Case counts obtained through comprehensive surveillance may be considered inherently variable even though they are essentially census-level quantities, in the sense that a case count can be viewed as representing one observation from a hypothetically repeatable process [4-7]. For relatively rare conditions such case counts are often assumed to follow a Poisson distribution [6,7]. For example, suppose that all M provisional cases were to be reviewed so that the count of true cases M_T _could be determined. When reporting the corresponding incidence rate R = M_T_/N one might also make use of the variance estimate , based on the assumption that M_T _represents one observation from a Poisson process [6,7]. Due to the estimation of PVP, however, the adjusted case count  cannot be treated in a similar fashion. Depending on the validation sample and the underlying PVP, for example, Var() can be well in excess of the variance that would be estimated under the assumption that  simply follows a Poisson distribution.

The remainder of this paper addresses three aspects of the problem outlined above: (i) a simple model for the true and false positive case counts within the defined framework, (ii) selected properties of  under a broadly applicable validation sample plan, and (iii) the relative frequency of coverage for interval estimates formulated using these properties.

### A Case Count Model

To evaluate the proposed estimator , a working model characterizing the process underlying the case counts M, M_T_, and M_F _is needed. For a given at-risk population and surveillance period it will be assumed that the provisional case count M is generated according to a Poisson process with parameter λ. Each provisional case, independent of other provisional cases, will be assumed to be a true case with probability equal to the underlying PVP. These assumptions are reflected in the following mixture model [8]:

M ~ POI(λ);

M_T_|M ~ BIN(M, PVP)

where POI denotes the Poisson distribution and BIN denotes the binomial distribution. The count of false positive cases is implicitly given by M_F _= M - M_T_. It is well-established that under this type of decomposition M_T _and M_F _are independent Poisson random variables such that M_T _~ POI(τ) and M_F _~ POI(φ), where τ = λ·PVP and φ = λ·(1-PVP) [9,10]. In this model, the parameter λ represents the average size of the recurring count of provisional cases and τ represents the average size of the recurring count of true cases among the provisional cases. The quantity 1/PVP can be viewed as the factor by which the count of true cases is inflated (on average) under the operational case definition. Finally, the parameters λ, τ and φ are implicitly dependent on the size of the at-risk population N; however, the functional form of this dependency is not important in the present development.

### A Validation Sample Plan

This section examines several important properties of the estimator  when a fixed fraction of provisional cases are sampled for confirmation. The properties presented are derived in Appendix A. Letting 0 < f < 1 denote the fixed sampling fraction, assume that the sample size S =  where the quantity f·M is rounded up. Under this procedure:

E[] = τ        (2)

and when f·λ is sufficiently large:



Equality (2) indicates that  is an unbiased estimator for the mean recurring count of true cases. The first component τ on the right-hand side of (3) represents the variance of the true case count M_T_. The second component approximates the addition to variance that results from the case count adjustment based on . Note that for any given PVP the variance inflation factor is essentially constant as a result of holding the sampling fraction fixed.

It is noted in passing that when case populations are typically small, it may be feasible to adopt the practice of confirming all provisional cases. Under this approach  will be equivalent to the true case count M_T _and it follows that  ~ POI(τ). Based on familiar properties of the Poisson distribution [8] it follows that E[] = Var() = τ and customary analysis methods are applicable.

### Application

The remaining objective is the formulation of a simple method for constructing interval estimates for τ and the corresponding incidence rate. From (2) it is already known that  is an unbiased estimator of τ. In Appendix B it is shown that the following estimator is nearly unbiased for the right-hand side of (3):



Based on (4) an approximate (1-α)·100% confidence interval (adjusted for the false positive bias) for the recurring case count τ is given by:



where z_α/2 _represents the appropriate quantile of the standard normal distribution. The corresponding interval estimate for the population-based incidence rate is:



where it will be recalled that N is the size of the at-risk population under surveillance. As an example, suppose that an interval estimate providing 95% relative frequency of coverage is desired for the population-based incidence rate. Table 1 shows the relative frequency with which interval (5) covers the underlying incidence rate in repeated Monte Carlo simulations involving various underlying values of PVP, λ, and f. For several cells f·λ is small and the coverage is below the nominal (95%) level, providing an illustration of where the interval estimation procedure begins to break down. In the remaining table cells coverage is close to the nominal level.

**Table 1:** Estimated Relative Coverage Frequencies of a Nominal 95% Interval with Variance Correction.

	PVP = 0.70			PVP = 0.80			PVP = 0.90		
f	λ = 100	λ = 500	λ = 1000	λ = 100	λ = 500	λ = 1000	λ = 100	λ = 500	λ = 1000
0.10	0.92	0.94	0.95	0.92	0.95	0.95	0.94	0.95	0.95
0.25	0.94	0.95	0.95	0.95	0.95	0.95	0.95	0.95	0.95
0.50	0.95	0.95	0.95	0.95	0.95	0.95	0.94	0.95	0.95

To illustrate the importance of the correction to the variance, Table 2 shows the relative coverage frequencies (again based on repeated simulations) if the adjusted case counts are simply assumed to follow a Poisson distribution. It is apparent that for smaller sampling fractions, coverage is well below the nominal level even with the larger case populations.

**Table 2:** Estimated Relative Coverage Frequencies of a Nominal 95% Interval w/o Variance Correction.

	PVP = 0.70			PVP = 0.80			PVP = 0.90		
f	λ = 100	λ = 500	λ = 1000	λ = 100	λ = 500	λ = 1000	λ = 100	λ = 500	λ = 1000
0.10	0.73	0.70	0.68	0.78	0.77	0.76	0.86	0.84	0.84
0.25	0.84	0.85	0.85	0.87	0.88	0.88	0.92	0.91	0.91
0.50	0.91	0.92	0.91	0.92	0.93	0.93	0.93	0.94	0.94

Extensions to independent subgroups (e.g., age groups) and aggregates (e.g., age-adjusted rates) are straightforward. Provided that subgroup boundaries do not divide the surveillance population too finely, the error associated with the interval estimation method described above should remain minimal.

## Conclusion

This paper was motivated by considerations related to analysis of data from the brain injury surveillance system mentioned in the introduction. Beginning with surveillance year 2000, a number of participating states identified provisional cases which were subsequently determined to be false positives upon in-depth review. Preliminary estimates of PVP were observed to fall close to 0.9 for some states, suggesting the need for adjusted incidence rate estimates. This issue is also relevant in a broader context, as a wide range of PVP estimates have been reported for other surveillance systems [11].

Adjustments to incidence rate estimates to eliminate the false positive bias are straightforward. However, since the PVP estimates used to make such downward adjustments are subject to random variation, the adjusted rates have an additional source of variation beyond what is usually assumed. Interval estimates failing to account for this fact may have coverage frequencies well below the nominal level. This paper presents a simple method of interval estimation for rates that have been adjusted to remove the bias due to false positives, applicable in large-scale surveillance settings.

The methodology presented does not address the potential bias associated with false negatives. In situations where validation data also support estimation of sensitivity, surveillance case counts could be further adjusted to reduce or eliminate such bias. This in turn would introduce another source of variation in the adjusted case counts and associated rates. Other types of sampling plans might also be considered. For example, a fixed sample size s* might be preferred, in which case S = min(s*, M) and an alternate expression for Var() would result. Technical details aside, the essential point is that data available from validation samples can have a nontrivial influence on point and interval estimates, and should be factored into surveillance statistics whenever feasible.

## Appendix A. Moments of the Estimator 

In the sampling procedure considered, the size of the validation sample depends on the provisional case count M. To make the analysis generic, the sample size will be denoted by s(M) where s(·) depends on the particular sampling procedure but is assumed positive whenever M > 0. The PVP-adjusted case count (1) can then be defined more precisely as:



where implicitly  = C_T_/s(M). When M > 0 the distribution of C_T _conditional on M and M_T _is hypergeometric [12], that is, C_T_|M, M_T _~ HYP(s(M), M_T_, M). It is not difficult to show that when M > 0 the distribution of C_T _conditional on M only is binomial, that is, C_T_|M ~ BIN(s(M), PVP). It follows that E[|M] = M·PVP for M ≥ 0. Applying principles of conditional expectation [8] it is readily established that  is an unbiased estimator of τ = λ·PVP:

E[] = E[E[|M]] = E[M·PVP] = λ·PVP.

To determine Var() it is convenient to employ the following variance decomposition [8]:

Var() = E[Var(|M)] + Var(E[|M]).

Since E[|M] = M·PVP it follows that Var(E[|M]) = λ·PVP^2^. Evaluation of the first component of variance is more complicated. Defining:



it follows from (A.1) and the fact that C_T_|M ~ BIN(s(M), PVP) when M > 0 that:

Var(|M) = PVP·(1-PVP)·g(M).

The task is thus reduced to determining E[g(M)]. When s(M) =  it holds that g(M) ≤ M/f and hence that E[g(M)] ≤ E[M/f] = λ/f. Given fixed f the upper bound is a good approximation provided that λ is sufficiently large, so that E[g(M)] ≌ λ/f and E[Var(|M)] ≌ PVP·(1-PVP)·λ/f. Combining variance components and simplifying results in:



Numerical calculation of Var() across a range of values for PVP, λ, and f shows that for f ≥ 0.01 and f·λ ≥ 50, the relative error of (A.2) is less than 0.01.

## Appendix B. An Estimate of Var()

The following is proposed as an estimator of the right-hand side of (A.2):



Defining:



it follows from the treatment in Appendix A that the expected value of the variance estimator (B.1) conditioned on M is:



Then, since  it follows that:



When s(M) =  it holds that h(M) ≤ 1/f and hence that E[h(M)] ≤ 1/f. Given fixed f the upper bound is a good approximation provided that λ is sufficiently large. Substituting 1/f in place of E[h(M)] results in:



Algebraic simplification results in:



As f·λ becomes large, approximation (A.2) results.

## Competing interests

The author(s) declare that they have no competing interests.
